# From spillover to systems: evidence gaps in One Health preparedness for emerging infectious diseases in Latin America and the Caribbean

**DOI:** 10.3389/fmed.2026.1938915

**Published:** 2026-08-26

**Authors:** Esteban Ortiz-Prado, María Paz Cadena, Jorge Vasconez-Gonzalez, Juan S. Izquierdo-Condoy

**Affiliations:** One Health Research Group, Universidad de las Américas, Quito, Ecuador

**Keywords:** climate change, emerging infectious diseases, integrated surveillance, Latin America, One Health, pandemic preparedness, zoonoses, zoonotic spillover

## Abstract

Latin America and the Caribbean are a global hotspot for emerging and re-emerging infectious diseases, yet regional One Health preparedness remains uneven and incompletely operationalized. This narrative Mini Review synthesizes evidence published mainly between 2015 and 2026 on One Health preparedness for emerging infectious diseases in the region, emphasizing how environmental disruption and climate change shape zoonotic and vector-borne spillover risk. Available regional surveys suggest broad professional familiarity with the One Health concept but limited operational implementation, with environmental health frequently identified as the least-integrated domain. We argue that spillover risk—and the failure to detect and contain spillover once it occurs—should be understood as a system-level outcome shaped by ecological disruption, socioeconomic vulnerability, surveillance capacity, and governance, rather than as an isolated biological event: deforestation, agricultural and extractive expansion—including illegal mining and logging—unplanned urbanization, and climate variability generate new human–animal–vector interfaces, while fragmented governance, uneven and poorly decentralized laboratory capacity, and limited reservoir and environmental surveillance leave these interfaces unmonitored. Environmental and climatic drivers are robustly linked to spillover, although the pathways are disease-specific rather than universal, and socioeconomic vulnerability concentrates the resulting burden in Indigenous, rural, and marginalized populations. We identify priority gaps in integrated surveillance, decentralized diagnostics, genomic capacity, reservoir ecology, governance, financing, and equity, and propose an agenda for anticipatory, climate-informed, and context-sensitive preparedness.

## 1 Introduction

Latin America and the Caribbean concentrate many of the ecological and social conditions that favor infectious disease emergence, including megadiverse tropical ecosystems, rapid land-use change, expanding agricultural and extractive frontiers, extensive urbanization accompanied by large informal settlements, and pronounced climate variability (1–3). Over the past decade, the region has experienced major arboviral epidemics, recurrent sylvatic yellow fever activity, the disproportionate effects of the COVID-19 pandemic, the emergence of mpox, and the southward expansion of highly pathogenic avian influenza A(H5N1) (4, 5). Although these events differ in their ecological origins and transmission dynamics, they have repeatedly exposed a common structural vulnerability: preparedness systems centered predominantly on human health and insufficiently connected to animal and environmental surveillance or to the ecological processes that shape infectious disease risk.

The One Health paradigm, which recognizes the interdependence of human, animal, and environmental health, provides a coherent framework for anticipating, detecting, and containing these threats (6, 7). The National Research Council (US) Committee on Achieving Sustainable Global Capacity for Surveillance and Response to Emerging Diseases of Zoonotic Origin proposed a five-stage framework describing the progression of animal pathogens from exclusive circulation in animals to sustained human-to-human transmission (8); Bernstein and colleagues subsequently emphasized spillover as an additional critical stage and advanced the concept of primary pandemic prevention: acting on the ecological and anthropogenic drivers of pathogen emergence before widespread transmission becomes established in human populations (9, 10). This perspective is particularly consequential for Latin America and the Caribbean because it shifts the focus of preparedness upstream—from predominantly reactive outbreak control toward the human–animal–environment interfaces where emergence begins. It also highlights that spillover risk, as well as failures to detect and contain cross-species transmission, is shaped by potentially modifiable factors such as land governance, ecosystem disruption, intersectoral coordination, socioeconomic vulnerability, and surveillance capacity. Spillover should therefore not be regarded solely as an unpredictable biological event, although not every cross-species transmission event can be prevented.

This Mini Review synthesizes peer-reviewed primary studies, systematic and scoping reviews, and regional analyzes published mainly between 2015 and 2026, drawing on a targeted search of the recent literature on One Health preparedness and the environmental and climatic drivers of infectious disease emergence in Latin America and the Caribbean. Earlier landmark publications were included when essential for conceptual framing. Because this is a narrative synthesis, the evidence presented is subject to selection and language biases, and country-level coverage is illustrative rather than exhaustive. We distinguish four interconnected but analytically separate stages: upstream ecological and social drivers; pathogen spillover at human–animal–environment interfaces; early detection and containment; and subsequent epidemic amplification. This distinction is important because the determinants, evidence gaps, and preparedness interventions relevant to each stage are related but not interchangeable. Accordingly, we first examine the environmental and climatic processes that create or intensify high-risk interfaces, then assess the current state of regional One Health preparedness, illustrate systemic challenges through selected pathogen systems, identify cross-cutting evidence gaps, and propose a research and policy agenda for anticipatory, climate-informed, and equity-oriented preparedness.

## 2 Environmental disruption, climate change, and the making of new interfaces

**Figure 1:**
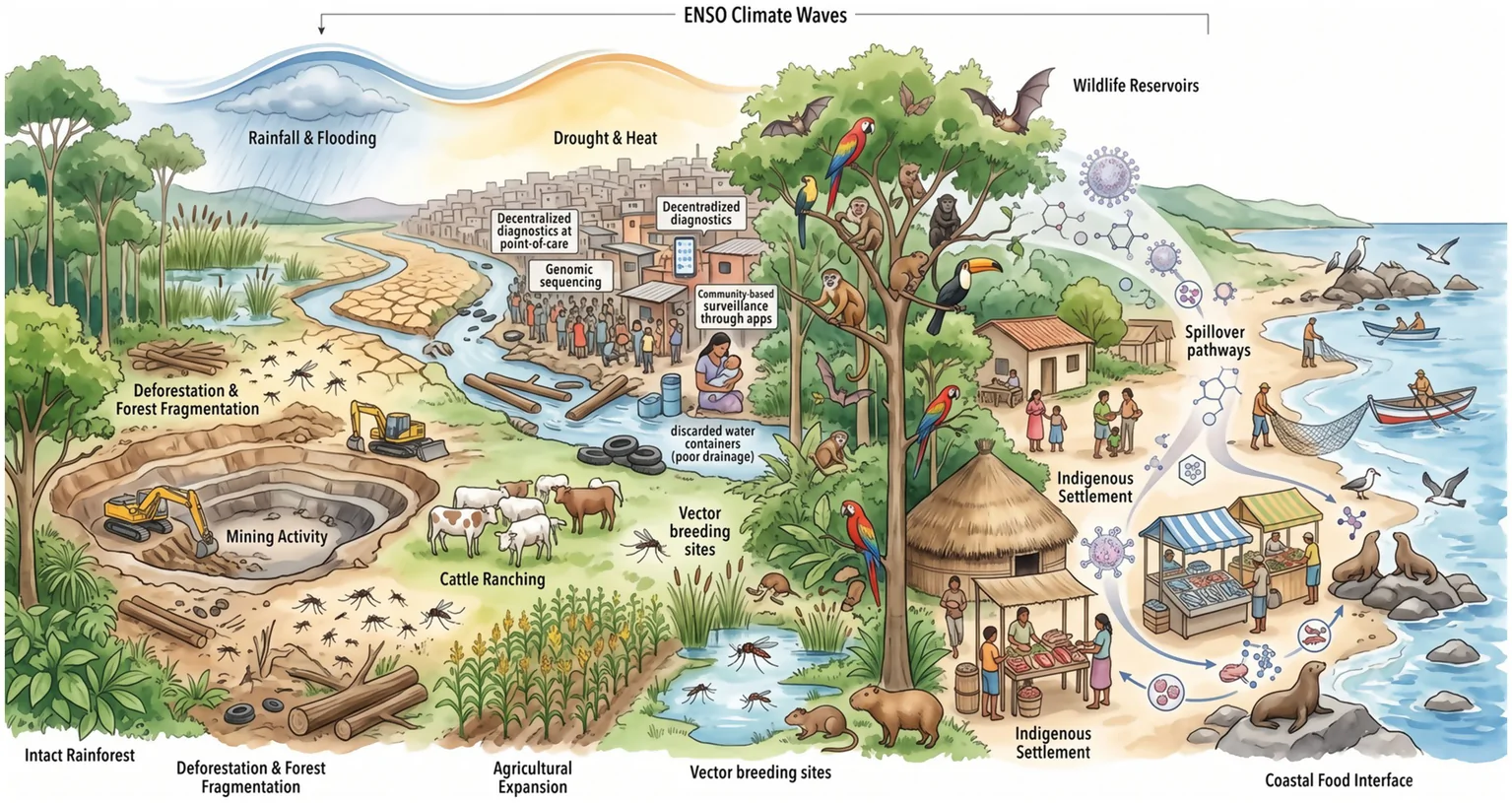
Environmental and climatic drivers of zoonotic and vector-borne disease emergence across a Latin American landscape gradient. The illustration depicts a continuum from intact rainforest through deforestation and forest fragmentation, mining, cattle ranching, agricultural expansion, and peri-urban informal settlements to coastal ecosystems. Climate variability associated with the El Niño–Southern Oscillation (ENSO)—rainfall and flooding, drought, and heat—modulates these drivers. Together they generate high-risk human–animal–vector interfaces, including wildlife reservoirs, vector breeding sites, Indigenous and rural settlements, and coastal food interfaces, through which spillover pathways connect wildlife, domestic animals, vectors, and human populations.

Zoonotic and vector-borne emergence in the region is driven less by pathogens appearing *de novo* than by anthropogenic changes that bring people, domestic animals, vectors, and wildlife reservoirs into new contact (11). Land-use change is a major proximate driver of zoonotic and vector-borne disease emergence in the region. In Brazil, loss of natural vegetation cover was the most important environmental predictor of neglected zoonotic disease probability, and the relationship was non-linear: risk was highest where ecosystem destruction was in its early stages, consistent with the concentration of spillover at fragmented forest edges (12). Landscape modeling further indicates that as native vegetation declines, mammalian communities may shift toward disturbance-tolerant generalist species, some of which can act as competent reservoirs (13). Importantly, this anthropogenic fingerprint is disease-specific: mosaic landscapes are a consistent predictor of vector-borne outbreak risk, whereas directly transmitted zoonoses do not show consistent associations with deforestation, underscoring the need for transmission-mode-specific risk frameworks (14) (Figure 1).

Climate change acts as both an amplifier of these drivers and a redistributor of risk into new geographic spaces, through disease-specific pathways. Phylodynamic analysis of the 2016–2021 Brazilian yellow fever epizootic linked higher temperatures to larger effective viral population sizes and faster spread, and associated dispersal with wetter, mosaic landscapes of high non-human-primate density (15, 16). For rodent-borne haemorrhagic fevers, species-distribution and force-of-infection modeling projects substantial increases in spillover risk for the New World arenaviruses (Guanarito, Machupo, Junín) under moderate and severe climate scenarios, with transboundary hotspots (17); hantavirus infections are consistently associated with precipitation and habitat type (18); and climate-driven rodent range expansion—projected at up to a 140% increase in suitable habitat by 2050 through Andean glacier retreat—raises the outbreak potential of the high-lethality Chapare arenavirus (19). Leptospirosis shows particularly clear climate signals, with incidence peaks aligned to El Niño–Southern Oscillation cycles and flooding (20, 21), and its ecological niche is expected to expand with rising temperature and humidity (22).

A distinctive feature of the region is the Amazonian wildlife–human interface. Hunting and consumption of wild meat, together with the keeping of non-traditional species, create direct pathways for spillover of pathogens ranging from *Mycobacterium leprae* in armadillos to enteric bacteria and diverse arboviruses (23). This interface is intensified by illegal extractive activities: unregulated gold mining and logging degrade and fragment forests, while the largely unvaccinated, highly mobile populations they draw into remote foci—together with the organized-crime and drug-trafficking networks that control access to many of these territories—experience heightened exposure to sylvatic pathogens and, at the same time, obstruct immunization and vector-surveillance campaigns (23, 24). Yellow fever exemplifies this dynamic: the 2025 South American outbreak 301 confirmed cases and 124 deaths (source-reported CFR, 41.1%) concentrated among unvaccinated populations at the forest–urban interface, was amplified by deforestation, forest fragmentation, and illegal mining, and was signaled by non-human-primate epizootics that act as ecological sentinels of viral circulation (24).

These heterogeneous processes converge on a small set of mechanisms: expansion of reservoir and vector populations, increased contact at forest edges and in peri-urban zones, reduction of host-diversity buffering, and pathogen amplification. Social ecology is inseparable from this picture—unplanned urbanization and irregular water supply amplify Aedes infestation in vulnerable neighborhoods (25), and socioeconomic variables predict transmission risk as strongly as environmental ones (12, 26). Human aggregation adds a further, episodic amplifier: large sporting events and mass gatherings, frequent across the region, concentrate people of diverse origins at high density and facilitate rapid person-to-person transmission and international admixture of respiratory and other pathogens, as illustrated by the mass celebrations following the 2022 FIFA World Cup in Argentina, which gathered millions and preceded a sharp rise in reported infections (27). Yet climate and these social amplifiers remain under-represented in regional surveillance and health information systems, limiting the translation of this evidence into anticipatory action (28, 29).

## 3 One Health preparedness in Latin America: concept, surveillance, operation, policy

Regional frameworks exist—including the Pan American Health Organization’s One Health orientation and established veterinary public-health programs (7, 30)—but implementation lags. In a convenience survey of 76 professionals from 13 countries—55% of whom were based in Colombia—respondents reported high familiarity with One Health but rated national implementation at a median of 3.0/5 and citizen awareness at 2.0/4, citing insufficient policymaker commitment, limited resources, and a siloed approach as the principal barriers (6). A regional survey of zoonoses programs found that 69% of respondents considered inter-ministerial relationships productive or very productive, whereas 31% described them as only minimally productive. Respondents nevertheless prioritized formal regional networks, improved communication, and integrated surveillance (31).

Structural fragmentation is most visible in the environmental pillar. A One Health Index applied to twelve South American countries found that better environmental health was not associated with better human or animal health, indicating that the three pillars operate in relative isolation (32). In a 2020 assessment of publicly available emergency-management plans from 86 countries, Latin America and the Caribbean had the lowest regional inclusion of animal health (33), a social-network analysis in Peru found collaboration concentrated between the human and agricultural sectors with weak environmental linkage and few connections between government levels (34), and reviews consistently identify absent or outdated legal frameworks, weak governance, and poor data interoperability as dominant barriers (30, 35).

Local and regional successes show what is achievable. A municipal One Health reform in Foz do Iguaçu, Brazil, integrating field teams, digital systems, and trained agents, produced an approximately ten-fold increase in zoonotic-disease notifications without impairing arbovirus surveillance—implying that conventional systems substantially underestimate true burden (36). At the regional scale, the ECLIPSE consortium provides cross-country surveillance for avian influenza, dengue, and antimicrobial resistance, though sectoral silos and funding constraints limit full operationalization (4). The prevailing pattern, however, is of preparedness that is conceptual and surveillance-oriented rather than operational and policy-embedded, with successes concentrated where motivated actors work within a narrow local scope rather than under mandated, financed national coordination (35).

## 4 Illustrative pathogen systems

The following systems are illustrative rather than exhaustive and were selected to represent three distinct preparedness challenges: wildlife and environmental sentinel surveillance, sylvatic–urban interface expansion, and climate- and vulnerability-mediated epidemic amplification.

### 4.1 Highly pathogenic avian influenza A(H5N1): the animal–environment blind spot

The expansion of H5N1 clade 2.3.4.4b across South America caused extensive mortality in wild birds and marine mammals. Genomic and epidemiological findings from marine-mammal outbreaks are consistent with mammal-to-mammal transmission and have identified mutations associated with mammalian adaptation, while sporadic human infections have also been documented in the region (5). The epizootic unfolded largely in the wildlife–environment domain—the region’s weakest surveillance sector—underscoring the sentinel value of wildlife mortality and the need for cross-border, One Health–integrated response, of which the ECLIPSE consortium is an early example (4, 5).

### 4.2 Oropouche virus: neglected arboviruses and ecological drivers

Oropouche virus is believed to circulate through sylvatic and urban transmission cycles. Viral detection and serological evidence have implicated sloths, non-human primates, and other vertebrates, although the definitive reservoir and sylvatic-vector roles remain incompletely characterized. Historically, more than 500,000 infections have been estimated in Brazil, although the true burden is likely higher because of limited diagnostic coverage and clinical overlap with other arboviral diseases (37). It exemplifies how ecological change, together with thin diagnostic coverage for “neglected” arboviruses routinely misclassified as dengue or Zika, permits silent geographic expansion (37, 38).

### 4.3 Urban arboviruses under climate stress: dengue, Zika, and chikungunya

Aedes-borne arboviruses remain the region’s dominant epidemic burden. A systematic review of Aedes ecology found that temperature and rainfall govern vector dynamics while urbanization and socioeconomic vulnerability amplify infestation by concentrating water-storage containers in areas with irregular supply (25). This system exemplifies the tight coupling of climatic and social drivers at the urban informal-settlement interface and the centrality of integrated vector management and entomological surveillance.

## 5 Cross-cutting evidence gaps

Across these systems, evidence gaps recur along predictable axes (Table 1). Animal and reservoir surveillance is the weakest link: most countries assessed globally lacked a functional wildlife-health surveillance program (39), and targeted bat metagenomics in São Paulo—repurposing existing rabies surveillance—recovered dozens of viral contigs across twelve families, including a previously unknown filovirus, illustrating the scale of uncharacterized reservoir diversity (40). Diagnostic incapacity compounds this, as mild febrile syndromes are routinely misclassified, producing systematic underestimation for pathogens such as West Nile virus and leptospirosis (38, 41). Genomic capacity and cross-sectoral data interoperability remain unequal (35); governance and financing deficits prevent sustained intersectoral operation (30); and burden is concentrated in Indigenous, Black, rural, and informal-settlement populations, often where veterinary and surveillance infrastructure is absent or where insecurity impedes it (12, 42, 43). Wildlife trade, hunting, and consumption remain among the highest-risk yet least-regulated interfaces (23, 44, 45), and regional evidence on the cost-effectiveness of preventive investment is sparse (9).

**Table 1:** Cross-cutting evidence gaps in One Health preparedness for emerging infectious diseases in Latin America, with rationale and suggested research or policy action.

Evidence-gap domain	Key gap	Why it matters	Research/policy action needed
Governance/policy	No mandated, financed intersectoral One Health body; environmental pillar least integrated; weak legal frameworks	One Health cannot move from concept to operation; lowest animal-health inclusion in emergency plans regionally	Legal mandates and national One Health units; sustained multisectoral financing; cross-border agreements
Animal/reservoir surveillance	Wildlife and livestock surveillance is the weakest sector; most countries lack functional wildlife-health programs	Spillover is missed at source; uncharacterized reservoir virome; NHP epizootics under-used as sentinels	Wildlife-mortality and NHP-epizootic sentinels; veterinary–public-health integration; reservoir viromics
Diagnostic/laboratory	Scarce decentralized diagnostics; misclassification of febrile syndromes as dengue/Zika	Delayed detection; systematic underestimation of burden (e.g., West Nile virus, leptospirosis)	Field-deployable assays; tiered laboratory networks; standardized serosurveys
Genomic/data systems	Sequencing capacity concentrated; poor data interoperability across sectors and levels	Blind spots for reassortment and host adaptation; fragmented decision-making	Sustained genomic networks; interoperable One Health data platforms
Ecological/environmental	Limited quantification of how land-use and climate change alter reservoir and vector distributions; disease-specific pathways	Predictive, transmission-mode-specific risk mapping is not possible	Longitudinal eco-epidemiological studies; remote sensing; climate-scenario modeling
Climate integration	Climate under-represented in surveillance and health information systems	Anticipatory, climate-informed response is precluded	Climate–health early-warning systems; co-production with local services
Health-system preparedness	Reactive, pathogen-specific systems; fragile financing; limited planning for mass gatherings	Anticipatory capacity is limited	All-hazards anticipatory preparedness; protected funding; mass-gathering preparedness; scaling of local successes
Equity/community	Burden concentrated in Indigenous, rural, and informal-settlement populations; veterinary absence and insecurity in remote frontier areas	Effectiveness and uptake are undermined; risk is invisible where surveillance and services are absent	Community-based surveillance; participatory, culturally appropriate programs; equity-centered immunization; rural veterinary capacity
Therapeutic/vaccine access	Inequitable access to vaccines and antivirals; import dependence	Response is delayed and inequitable	Regional manufacturing and health sovereignty; strategic stockpiles
Economic/cost-effectiveness	Sparse regional evidence on cost-effectiveness of One Health and primary prevention	The investment case is weakened	Regional economic evaluations incorporating climate and biodiversity co-benefits

## 6 Discussion: from spillover to systems a research and policy agenda

The evidence supports reframing preparedness around the interfaces where emergence begins. Primary prevention at source—reducing deforestation, curbing illegal mining and logging, regulating wildlife trade and improving husbandry biosecurity, and strengthening the health and economic security of communities in emergence hotspots—has been estimated to cost a small fraction of the losses from zoonotic pandemics while yielding climate and biodiversity co-benefits (9, 10). For a region whose emergence hotspots overlap with globally significant forests, this argument aligns pandemic prevention with existing climate and conservation commitments. Because the ecological pathways are disease-specific, prevention strategies must likewise be tailored by transmission mode rather than assuming a single universal mechanism (14).

**Figure 2:**
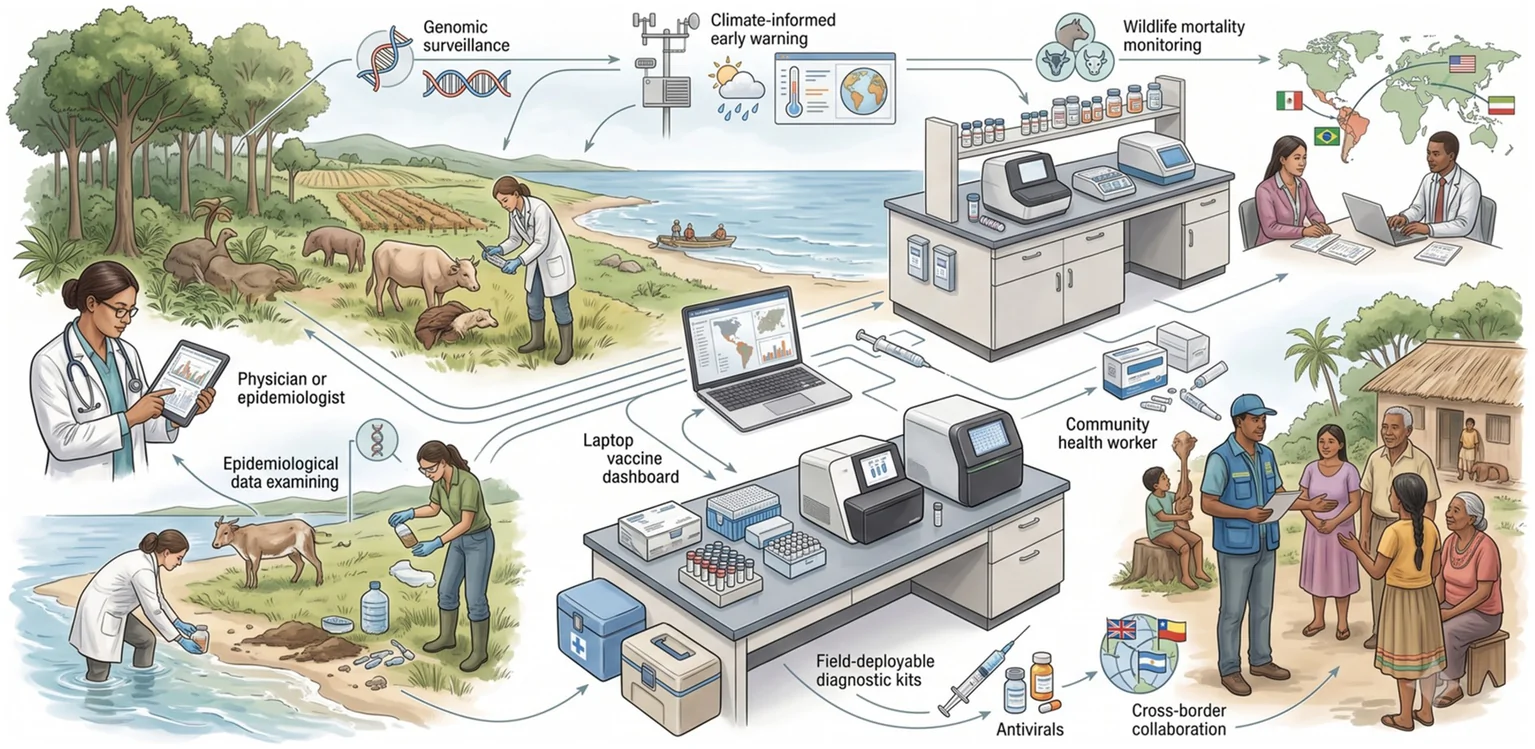
Components of an integrated One Health preparedness system for emerging infectious diseases in Latin America. The schematic links field, laboratory, and community activities across the human–animal–environment interface, including genomic surveillance, climate-informed early warning, wildlife-mortality monitoring, veterinary and environmental sampling, epidemiological data analysis, decentralized and field-deployable diagnostics, vaccine and antiviral deployment, community health-worker engagement, and cross-border collaboration. Arrows indicate the flow of information and specimens from surveillance and diagnosis toward a coordinated, equity-oriented public health response.

Operationalizing One Health beyond rhetoric requires several concurrent shifts (Figure 2): (i) legally mandated, financed intersectoral governance rather than *ad hoc* committees (30); (ii) integrated human–animal–environmental surveillance incorporating wildlife, non-human-primate epizootic, and reservoir streams and interoperable data systems (24, 39, 40); (iii) decentralized diagnostic and genomic capacity extended to remote, border, island, Amazonian, and Andean territories and sustained beyond emergency funding (35, 38); (iv) climate-informed early-warning systems linking meteorological and ENSO indicators to vector and reservoir dynamics (28, 29); (v) equitable access to diagnostics, vaccines, and antivirals, with equity-centered immunization at the forest–urban interface, restored rural veterinary capacity, and meaningful community and Indigenous engagement (12, 24, 42); and (vi) cross-border surveillance and data-sharing, together with anticipatory planning for mass gatherings and major sporting events, given the transboundary movement of pathogens such as H5N1 and the arenaviruses and the episodic amplification associated with large-scale human aggregation (4, 17, 27). The research priorities follow from the gaps in Table 1: quantifying spillover risk across land-use gradients, modeling reservoir and vector distributions under climate scenarios, validating field-deployable diagnostics, and conducting regional economic evaluations of preventive investment in a demonstrably changing risk landscape (46). Scaling documented local successes, such as the Foz do Iguaçu model, offers a pragmatic pathway (36).

Several limitations apply. This is a narrative synthesis; geographic coverage is illustrative; several cited analyzes are recent preprints whose peer-reviewed versions should be substituted where available; and the apparent scarcity of evidence in some domains may partly reflect publication and language bias rather than a true absence of regional activity.

## 7 Limitations

This review has several limitations. First, it is a narrative rather than systematic synthesis and did not include duplicate screening, formal risk-of-bias assessment, or quantitative evidence grading. Second, the regional literature is unevenly distributed, with substantial representation from Brazil and more limited evidence from several Central American and Caribbean countries. Third, definitions and operational indicators of One Health implementation vary substantially across studies. Fourth, selected preprints were included where peer-reviewed evidence was unavailable, and their findings should be interpreted cautiously. Finally, publication and language biases may cause documented evidence gaps to overstate the true absence of local or governmental activity.

## 8 Conclusion

Latin America’s exposure to emerging infectious diseases is inseparable from its ecological transformation and climate vulnerability. Treating spillover as a system-level failure reframes preparedness as an upstream, cross-sectoral task rather than a purely clinical or reactive one. Realizing this shift will require integrated, anticipatory, climate-informed, and equity-oriented One Health systems—supported by mandated intersectoral governance, sustained financing, decentralized diagnostic and genomic capacity, strengthened reservoir and environmental surveillance, and genuine community engagement—so that the region can move from predominantly reactive outbreak response toward reducing spillover risk, detecting cross-species transmission earlier, and containing emerging threats before sustained epidemic amplification occurs.
